# *Centella asiatica* (L.) Urban: From Traditional Medicine to Modern Medicine with Neuroprotective Potential

**DOI:** 10.1155/2012/946259

**Published:** 2012-05-14

**Authors:** Ilkay Erdogan Orhan

**Affiliations:** ^1^Department of Pharmacognosy, Faculty of Pharmacy, Gazi University, 06330 Ankara, Turkey; ^2^Department of Pharmacognosy and Pharmaceutical Botany, Faculty of Pharmacy, Eastern Mediterranean University, Gazimagosa, (Famagusta), Cyprus

## Abstract

This paper covers the studies relevant to neuroprotective activity of *Centella asiatica* (L.) Urban, also known as “Gotu Kola.” The plant is native to the Southeast Asia and has been used traditionally as brain tonic in ayurvedic medicine. The neuroprotective effect of *C. asiatica* has been searched using the key words “*Centella, Centella asiatica, gotu kola, Asiatic pennywort, neuroprotection, and memory*” through the electronic databases including Sciencedirect, Web of Science, Scopus, Pubmed, and Google Scholar. According to the literature survey, *C. asiatica* (gotu kola) has been reported to have a comprehensive neuroprotection by different modes of action such as enzyme inhibition, prevention of amyloid plaque formation in Alzheimer's disease, dopamine neurotoxicity in Parkinson's disease, and decreasing oxidative stress. Therefore, *C. asiatica* could be suggested to be a desired phytopharmaceutical with neuroprotective effect emerged from traditional medicine.

## 1. Introduction

**Figure 1:**
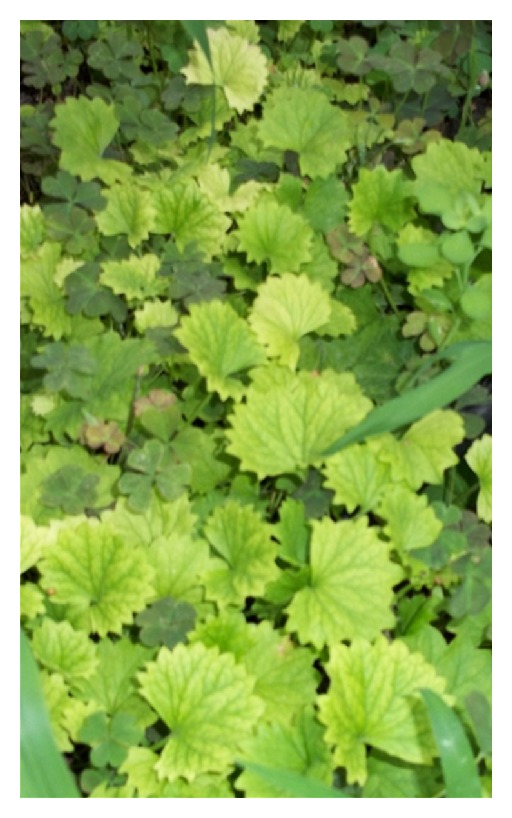
*Centella asiatica* (L.) Urban (Apiaceae).

*Centella asiatica* (L.) Urban (*Syn*. *Centella coriacea* Nannfd., *Hydrocotyle asiatica* L., *Hydrocotyle lunata* Lam., and *Trisanthus cochinchinensis* Lour.) is a tropical medicinal plant from Apiaceae family native to Southeast Asian countries such as India, Sri Lanka, China, Indonesia, and Malaysia as well as South Africa and Madagascar [1]. *C. asiatica*, commonly known as “Gotu kola, Asiatic pennywort, Indian pennywort, Indian water navelwort, wild violet, and tiger herb” in English, is a tropical plant, which has been also cultivated successfully due to its medical importance in some countries including Turkey, and it has a long history of utilization in ayurvedic and Chinese traditional medicines since centuries [2]. The leaves, which are edible, are in yellowish-green color, thin, alternate with long petioles, and quite characteristic reniform, orbicular, or oblong-elliptic shapes with seven veins [3] (Figure 1). The plant grows horizontally through its green to red stolones which combine to each other and roots in underground. Monographs of the plant describing mainly its wound healing and memory enhancement effects exist in the European Pharmacopeia, Commission E of the German Ministry of Health, and World Health Organization (WHO) [4]. In addition to neuroprotective effect of *C. asiatica*, it has been reported to own a wide range of biological activities desired for human health such as wound healing [5–7], anti-inflammatory [8, 9], antipsoriatic [10], antiulcer [11, 12], hepatoprotective [13], anticonvulsant [14], sedative [15], immunostimulant [16], cardioprotective [17, 18], antidiabetic [19], cytotoxic and antitumor [20, 21], antiviral [22], antibacterial [23], insecticidal [24], antifungal [25], antioxidant [26–28], and for lepra [29] and venous deficiency treatments [30, 31].

 Numerous preparations of this plant in various pharmaceutical forms recommended for several indications including neurological disorders are available allover the world. Taking this fact into consideration, many researchers have focused on neuroprotective effect of *C. asiatica* in order to confirm its traditional use on scientific base. For this purpose, a literature survey has been performed using the databases searched up to the year 2012 for the latest information on *C. asiatica*. This paper aims to cover up *in vitro*, *in vivo*, and clinical studies reporting the results relevant to neuroprotective effect of this plant.

## 2. Phytochemical Content of *C. asiatica*

**Figure 2:**
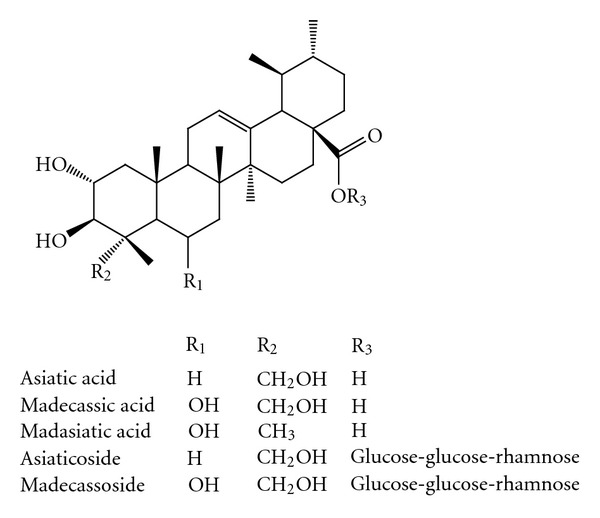
The major triterpene saponoside derivatives found in *Centella asiatica*.

*C. asiatica* has been reported to contain a vast number of compounds belonging to different chemical classes. The major chemical class found in this plant is triterpene saponosides. The major ones are known as asiatic acid, madecassic acid (6-hydroxy-asiatic acid), asiaticoside, madecassoside, and madasiatic acid (Figure 2), betulinic acid, thankunic acid, and isothankunic acid [32, 33]. Moreover, There are some other triterpenes such as brahmic acid, centellin, centellicin, asiaticin, bayogenin, terminolic acid, 3**β**,6**β**,23-trihydroxyolean-12-en-28-oic acid, 3**β**,6**β**,23-trihydroxyurs-12-en-28-oic acid, 3-O-[**α**-L-arabinofuranosyl] 2**α**,3**β**,6**β**,23-**α** tetrahydroxyurs-12-en-28-oic acid, centellasapogenol A, centellasaponins A-D, ursolic acid, pomolic acid, 3-epimaslinic acid, 23-O-acetylmadecassoside, and 23-O-acetylasiaticoside B [34–41].

**Figure 3:**
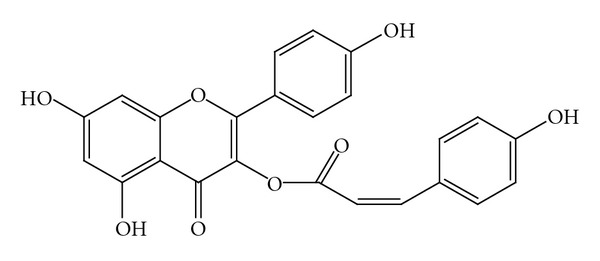
Castilliferol.

**Figure 4:**
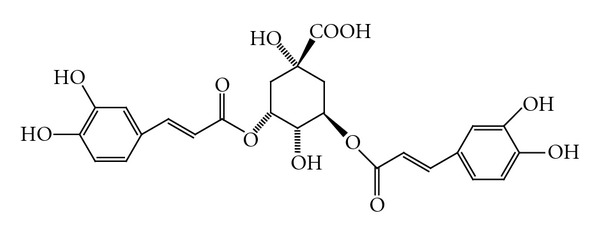
Isochlorogenic acid.

Presence of several flavonoid derivatives such as quercetin, kaempferol, patuletin, rutin, apigenin, castilliferol (Figure 3), castillicetin, and myricetin has been reported in *C. asiatica* [35, 39, 42], while isolation of polysaccharides (e.g., centellose) [43], polyacetylenes (e.g., cadinol, acetoxycentellinol, centellin, centellicin, and asiaticin) [36, 44], sterols (e.g., 11-oxoheneicosanil-cyclohexane, dotriacont-8-en-1-oic acid, sitosterol 3-O-**β*-*glucoside, stigmasterol 3-O-**β**-glucoside, and castasterone) [41, 45, 46], and phenolic acids (e.g., rosmarinic acid, 3,5-di-O-caffeoil quinic acid, 1,5-di-O-caffeoil quinic acid, 3,4-di-O-caffeoil quinic acid, 4,5-di-O-caffeoil quinic acid, ettacrynic acid, chlorogenic acid, and isochlorogenic acid [Figure 4]) [40, 42, 47] has been also identified in this species. In our quantitative study on *C. asiatica* of Turkish origin by HPLC, we reported existence of several phenolic acids, for example, *p*-hydroxybenzoic acid, vanillic acid, *p*-coumaric acid, *o*-coumaric acid, and *trans*-cinnamic acid [48].

On the other hand, only a few studies have described the chemical composition of the essential oils obtained from *C. asiatica* from Japan, South Africa, and Thailand, which mainly consisted of monoterpene and sesquiterpene derivatives [49–51]. In our work, we examined the essential oil composition of *C. asiatica* cultivated in Turkey by GC-MS for the first time and identified *α*-copaene as the major component [48].

## 3. Neuroprotective Activity of *C. asiatica*

### 3.1. *In Vitro* Studies

*C. asiatica* (gotu kola) is a reputed plant species for its traditional use in ayurvedic and Chinese medicines [52], and its positive effects on brain aging have been generally attributed to its two major triterpene saponosides; asiatic and madecassic acids as well as their heterosides; asiaticoside and madecassoside, respectively. For instance, the hydroalcoholic extract of the plant was tested *in vitro* against acetylcholinesterase (AChE), the key enzyme taking a critical role in the pathogenesis of Alzheimers disease (AD). Since deficit in the level of acetylcholine (ACh), which is hydrolyzed by AChE, has been identified in the brains of AD patients, inhibition of AChE as well as its sister enzyme butyrylcholinesterase (BChE) has become a rational target in drug development against AD [53]. The extract was found to inhibit AChE with 50% of inhibition rate at 150 *μ*g/mL concentration by the spectrophotometric method of Ellman [54]. In our study on the ethanol extracts prepared from the aerial parts of *C. asiatica* of both Turkish and Indian origins along with the standardized gotu kola extract (containing 10.78% of total asiaticoside and madecassoside) imported from China, we comparatively examined inhibitory potential of these three extracts against AChE, BChE, and tyrosinase (TYRO) at 50, 100, and 200 *μ*g/mL concentrations [48]. As aforementioned that cholinesterases are the important enzymes for AD treatment, TYRO has become an important target for Parkinson's disease (PD) since this enzyme plays a role in neuromelanin formation in the human brain and could be significant in occurrence of dopamine neurotoxicity associated with neurodegeneration linked to PD [55]. According to our results obtained at 200 *μ*g/mL, only the standardized extract was found to inhibit AChE (48.28 ± 1.64%), whereas the ethanol extracts of the plant samples from Turkey and India exerted 46.95 ± 0.94% and 70.30 ± 3.77% against BChE, respectively, and a notable inhibition against TYRO (42.83 ± 4.21% and 56.20 ± 3.17%, resp.).

 Awad et al. investigated inhibitory property of *C. asiatica* extract towards glutamic acid decarboxylase (GAD) and **γ**-aminobutyric acid transaminase (GABA-T), which are the enzymes responsible for GABA metabolism and found out that the extract stimulated the activity of GAD over 40% [56]. On the other hand, the leaf extract of *C. asiatica* growing in China was shown to display neuroprotection through enhancing phosphorylation of cyclic AMP response element binding protein (CREB) in neuroblastoma cells in A*β*(1–42) proteins found within the amyloid plaques occurring in the brains of AD patients [57]. In another study [58], effect of the aqueous leaf extract of the plant on monomers or oligomers that lead to formation of A*β*(1–42) proteins in AD *via* aggregation was examined using both thioflavin-T test and transmission electron microscope; however, it was observed not to cause any inhibition on aggregation of the monomers and oligomers. Inhibitory activity of the aqueous extract of *C. asiatica* that contained 84% of asiaticoside was tested by the radioenzymatic assay against phospholipase A_2_ (PLA_2_), which play role in neuropsychiatric diseases. The findings pointed out to the fact that the extract could inhibit Ca^2+^-independent PLA_2_ and cytosolic PLA_2_ [59]. The ethanol extract of the plant was observed to cause an increase in neurite development in human SH-SY5Y cell lines at 100 *μ*g/mL concentration, whereas its aqueous extract did not lead any increase in the same cells [60]. Then, the subfractions of the ethanol extract were also tested further in the same assay for neuritic development, and the most effective subfraction was demonstrated to have a nonpolar chemical nature. According to the results of that study, the authors concluded that *C. asiatica* extract might be beneficial in prevention of neuronal damage.

 Lee et al. studied neuroprotective potential of thirty six derivatives of asiatic acid prepared by various structural modifications and tested in primary cell culture consisting of rat cortical neurons exposed to glutamate, which is known as a neurotoxin [61]. Three of the compounds displayed higher protective activity than asiatic acid *per se* and also significantly reduced production of glutamate-induced nitric oxide (NO) as well as levels of glutathione, glutathione peroxidase, and some other related enzymes.

### 3.2. *In Vivo* Studies

 Neuroprotective effect of *C. asiatica* and its major triterpene saponosides has been extensively studied through different experimental models on animals such as passive avoidance and elevated-plus labyrinth tests for memory enhancing effect [62]. A research was carried out in rats to determine effect of the aqueous extract of *C. asiatica* on intracerebrovascular streptozocin-induced memory associated with sporadic type of AD by applying the extract at doses of 100, 200, and 300 mg/kg (b.w.) and measuring some oxidative stress parameters such as glutathione, superoxide dismutase (SOD), and catalase (CAT) [63]. While a clear dose-dependent improvement was observed in memory-related behaviors in the rat group administered the extract at 200 mg/kg (b.w.) dose, a serious decrease in malondialdehyde (MDA) and an increase in glutathione and CAT levels were recorded, which led to a final suggestion by the authors that *C. asiatica* extract has a positive effect on memory that is also related to its remarkable antioxidant effect. The same research group subjected this extract to passive avoidance and spontaneous locomotor activity behavioral tests using pentylenetetrazole-(PTZ-) induced memory loss in rats at 100 and 300 mg/kg (b.w.) doses [64]. Following the behavioral tests, MDA and glutathione levels were determined in the rat brains as oxidative stress markers, which significantly contribute to neurodegeneration. Accordingly, the extracts at the tested doses caused a notable improvement in all test parameters.

 In another study by Rao et al. [65], enhancing effect of *C. asiatica* extract on learning and memory was examined during 15 days at 200, 500, 700, and 1000 mg/kg (b.w.) doses by oral administration to mice. Open area, light/dark compartment, and radial-armed labyrinth tests were applied as experimental models, while AChE activity and dendritic arborization development were taken into consideration as biochemical markers. According to the findings, the extract displayed improving effect in radial-armed labyrinth test, whereas it did not cause any change in locomotor activity. On the other hand, extract administration resulted in an increase in AChE activity and dendritic arborization in CA3 neurons located in hippocampus. Thus, the authors concluded that the extracts may positively influence neuronal morphology, especially in young adult mice. In a similar study performed by the same researchers, the fresh leaf extract of *C. asiatica* was given to adult mice at 2, 4, and 6 mL/kg doses during 2, 4, and 6 weeks, respectively [66]. After these periods, the removed brains of mice were investigated under microscope, which pointed out to the evidence that the extract given at 6 mL/kg dose during 6 weeks caused a significant augment in dendritic arborization in neurons. These authors came to another similar conclusion that the juice obtained by pressing the fresh leaves of *C. asiatica* tested in the same experimental model in mice also enhanced dendritic arborization [67]. Besides, *C. asiatica* extract was shown to reduce levels of **β**-amyloid plaques in hippocampus in mice [68].

 Shinomol and Muralidhara investigated effect of *C. asiatica* extract against oxidative stress and mitochondrial dysfunction induced by 3-nitropropionic acid, a fungal-derived neurotoxin, in the brains of male prepubertal mice, and the extract was found to diminish oxidative stress remarkably through influencing the parameters such as MDA and radical oxygen species [69]. In a related study on rats, *C. asiatica* extract was reported to have a protective effect against mitochondrial damage occurred in PD by means of improving oxidative stress parameters [70]. Anticonvulsant effect of the crude material and extracts prepared from *C. asiatica*, also known as “brahmi” in Hindu, was determined in PTZ-induced convulsion model in rats and compared with fenitoin as the reference drug [71]. The data indicated that the crude material of the plant exerted a mild level of anticonvulsant effect at 500 mg/kg dose, while the methanol extract had superior effect to that of the crude material at 3rd and 6th hs. The extract prepared with propylene glycol also produced a dose-dependent anticonvulsant activity at 500 and 1000 mg/kg (b.w.) doses. Similarly, Ganachari et al. demonstrated *in vivo* anticonvulsant effect of the hydroalcoholic extract of *C. asiatica* against PTZ- and strychnine-induced opistotonus convulsions at 100 mg/kg (b.w.) [72]. Moreover, the extract was observed to reduce lipid peroxidation and spontaneous locomotor activity, whilst it potentiated pentobarbital-induced sleeping duration and diazepam-induced hyperactivity. In another paper [73], the ethyl acetate fraction of *C. asiatica *as well as combination of the fraction with some antiepileptic drugs including fenitoin, valproate, and gabapentin individually was administered intraperitoneally to the mice with convulsion induced by PTZ and found that the combinations caused an additive effect producing a higher anticonvulsant activity than each of the drugs. Additionally, neurotoxicity of the fraction and each combination was established by rotarod test, and combination of the extract with gabapentin was less neurotoxic. In the light of this evidence, the authors stated that conjoint use of the ethyl acetate fraction of *C. asiatica* with epileptic drugs might be beneficial for epileptic patients. In another study [74], De Lucia et al. reported anticonvulsant and sedative activities of the hydroalcoholic extract of *C. asiatica* in rats using elevated-plus labyrinth and PTZ-induced convulsion models, and the extract was also shown to exert low toxicity by chronic application with the LD_50_ value of 675 mg/kg (b.w.). Anticonvulsant activity of the hexane, chloroform, ethyl acetate, water, and *n*-butanol extracts prepared from *C. asiatica* was determined using PTZ-induced convulsion model in male Wistar rats, and effect of the extracts was also searched on Na^+^/K^+^, Mg^2+^, and Ca^2+^-ATPase activity [75]. The results pointed out to an increase in activity of three types of ATPases in the extract-administered groups accompanied by anticonvulsant activity. Anxiolytic activity of the hexane, ethyl acetate, and methanol extracts of *C. asiatica* and asiaticoside was tested using elevated-plus labyrinth, open area, social interaction, locomotor activity, and new cage models in rats [76]. The results indicated that only the methanol and ethyl acetate extracts of the plant along with asiaticoside displayed anxiolytic activity in elevated-plus labyrinth test. In another paper [77], sedative effect of *C. asiatica* was mainly attributed to brahmoside and brahminoside, the triterpene derivatives, whereas anxiolytic activity was suggested to be partly resulted from the interaction with cholecystokinin receptors (CCK_B_), a group of G protein-coupled receptors which are considered to take a potential place in modulation of anxiety, nociception, and memory.

*C. asiatica* extract was administered orally to old rats during 60 days at 300 mg/kg (b.w.) dose *per* day, and the cortex, hypothalamus, striatum, cerebellum, and hippocampus regions of the rat brains were investigated in terms of lipid peroxidation and protein carbonyl (PCO) contents [78]. The researchers made a statement that the extract may be showing a neuroprotective effect in old rats by way of bringing about a significant decrease in PCO contents and lipid peroxidation. Radical scavenging effect of the chloroform-methanol (4 : 1) extract of the plant was examined in monosodium glutamate-treated Sprague-Dawley female rats at 100 and 200 mg/kg doses [79]. Following the extract administration, a significant increase was observed in SOD and CAT levels, whereas glutathione level was not influenced. Flora and Gupta reported that the flavonoid fraction of *C. asiatica* demonstrated a protecting effect against lead acetate-induced neurotoxicity in mice through antioxidant mechanisms [80]. In another paper, asiatic acid, one of the major triterpene derivatives in *C. asiatica*, administered orally at 30, 75, and 165 mg/kg (b.w.) doses, was shown to have neuroprotective property in mice with permanent cerebral ischemia by evaluating infarct volume and behavioral changes between 1st and 7th days [81]. In the same study, the compound was additionally investigated in HT-22 cells exposed to oxygen glucose in terms of cell viability and mitochondrial membrane potential. Asiatic acid considerably diminished the infarct volume by 60% and 26% at the 1st and 7th days, respectively, which improved neurological status at 24 h after ischemia. The authors concluded that asiatic acid, which might be mediated to some extent by decreasing blood-brain barrier permeability as well as reduction in mitochondrial damage, could be useful for cerebral ischemia treatment.

 Probable improving effect of *C. asiatica* extract at 150 and 300 mg/kg (*p.o.*) doses was assessed against colchicine-induced memory using Morris water maze and plus-maze performance tests in male Wistar rats as well as oxidative damage parameters such as lipid peroxidation, nitrite, reduced glutathione, glutathione-S-transferase, SOD, and as a biochemical parameter, AChE activity [82]. The 25-day chronic administration of the extract caused a significant improvement in memory and oxidative damage parameters along with AChE activity. On the other hand, asiaticoside from *C. asiatica* exerted a neuroprotective effect against PD by reversing neurotoxicity induced by 1-methyl-4-phenyl-1,2,3,6-tetrahydropyridine (MPTP) in rats *via* balancing dopamine and antioxidant mechanism [83].

 Antidepressant activity of *C. asiatica* was evaluated using its triterpene fraction in cortex, hippocampus, and thalamus regions of rat brains by determining the corticosterone levels [84]. The triterpene fraction created a momentous diminution in corticosterone level and a notable increase in amount of monoamine-related neurotransmitters.

### 3.3. Clinical Studies

 Although many *in vivo* studies have been carried out on central-nervous-system-(CNS-) related effects of *C. asiatica*, the literature survey has revealed presence of only a limited number of clinical studies with this species. The results of an early double-blind clinical study on the children with mental deficiency in 1977 showed that a statistically significant improvement was recorded in the children in 3rd and 6th months following administration of *C. asiatica* [85].

 Possible effect of the capsulated aqueous extract of *C. asiatica* standardized to contain 29.9 mg/g tannic acid, 1.09 mg/g asiaticoside, and 48.89 mg/g asiatic acid was determined in a randomized, double-blind, and placebo-controlled clinical study carried out on 28 healthy and elder volunteers consisting of 4 men and 24 women with the average age of 65.05 ± 3.56 in Thailand [86]. The extract was given to the subjects once a day at 250, 500, and 750 mg doses during 2 months, and their cognitive performance was evaluated by a variety of parameters using computer-assisted techniques. The findings revealed that the highest dose of *C. asiatica* extract tested in this study possessed a cognitive enhancing effect. In a similar study [87], Dev et al. investigated effect of the capsulated *C. asiatica* extract on cognitive performance conducted with a total 41 of middle-age healthy subjects consisting of 22 women and 19 men. The extract was given to the subjects in a capsule once a day during 2 months. The cognitive performance was measured using Woodcock-Johnson Cognitive Abilities Test III (WJCAT III), and the extract was found to have a remarkably positive influence on all of the subjects. A recent clinical study consisting of 60 elderly subjects with average age of 65 with mild cognitive deficiency indicated that *C. asiatica* extract administered at 500 mg dose twice *per* day during 6 months led to a significant cognitive improvement according to Mini Mental State Examination (MMSE) scoring [88].

## 4. Precautions

 Although *C. asiatica* is one of the top-selling herbal medicines due to its remarkable pharmacological effects, some precautions should be taken for this plant. It has been known to be safe when taken at the recommended doses; however, skin irritation and contact dermatitis have been reported in some cases [89–91]. In a very early paper in 1969 [92], the total saponoside fraction containing brahmic acid and its derivatives of the plant was stated to cause infertility in an experiment conducted on human and rat sperms. In consistency with this paper, Newall et al. also affirmed that infertility was observed in female mice after oral administration of *C. asiatica* [93]. Another result pointed out to the fact that chronic treatment of *C. asiatica* might induce a spontaneous abortion in pregnant women [94]. Since the plant may bring about a raise in blood sugar and lipid levels, diabetic and hyperlipidemic patients should consider taking preparations of *C. asiatica* [93]. Briefly, maximum duration suggested for the use of *C. asiatica* preparations is 6 weeks, and at least, a 2-week break is needed after every long duration use. Even though no drug interaction has been reported for this plant up to date, pregnant and breast-feeding women are suggested to avoid using this herbal medicine.

## 5. Conclusion

*C. asiatica*, widely known as “gotu kola,” is a reputed medicinal plant for its various pharmacological effects favorable for human health. Besides its potent wound healing property, a number of studies described the noteworthy protective effect of the plant against several diseases of CNS. Biological effects of *C. asiatica* have been generally attributed to the major triterpene derivatives including asiatic acid, madecassic acid, asiaticoside, madecassoside, and brahmic acid. The neuroprotective effect of the plant has been suggested to result from different mechanisms, most of which have referred to positive influences on oxidative stress parameters.

## References

[1] Jamil SS, Nizami Q, Salam M (2007). *Centella asiatica* (Linn.) Urban: a review. *Natural Product Radiance*.

[2] Meulenbeld GJ, Wujastyk D (2001). *Studies on Indian Medical History*.

[3] Chopra RN, Nayar SL, Chopra IC (1986). *Glossary of Indian Medicinal Plants (Including the Supplement)*.

[4] Howes MJR, Houghton P (2003). Plants used in Chinese and Indian traditional medicine for improvement of memory and cognitive function. *Pharmacology Biochemistry and Behavior*.

[5] Tenni R, Zanaboni G, De Agostini MP, Rossi A, Bendotti C, Cetta G (1988). Effect of the triterpenoid fraction of Centella asiatica on macromolecules of the connective matrix in human skin fibroblast cultures. *Italian Journal of Biochemistry*.

[6] Suguna L, Sivakumar P, Chandrakasan G (1996). Effects of *Centella asiatica* extract on dermal wound healing in rats. *Indian Journal of Experimental Biology*.

[7] Shetty BS, Udupa SL, Udupa AL (2008). Biochemical analysis of granulation tissue in steroid and *Centella asiatica* (Linn) treated rats. *Pharmacologyonline*.

[8] Somchit MN, Sulaiman MR, Zuraini A (2004). Antinociceptive and antiinflammatory effects of *Centella asiatica*. *Indian Journal of Pharmacology*.

[9] George M, Joseph L, Ramaswamy (2009). Anti-allergic, anti-pruritic, and anti-inflammatory activities of *Centella asiatica* extracts. *African Journal of Traditional, Complementary and Alternative Medicines*.

[10] Sampson JH, Raman A, Karlsen G, Navsaria H, Leigh I (2001). *In vitro* keratinocyte antiproliferant effect of *Centella asiatica* extract and triterpenoid saponins. *Phytomedicine*.

[11] Cheng CL, Koo MWL (2000). Effects of *Centella asiatica* on ethanol induced gastric mucosal lesions in rats. *Life Sciences*.

[12] Cheng CL, Guo JS, Luk J, Koo MWL (2004). The healing effects of *Centella* extract and asiaticoside on acetic acid induced gastric ulcers in rats. *Life Sciences*.

[13] Pingale SS (2008). Evaluation of effect of *Centella asiatica* on CCL_4_ induced rat liver damage. *Pharmacologyonline*.

[14] Sudha S, Kumaresan S, Amit A, David J, Venkataraman BV (2002). Anti-convulsant activity of different extracts of *Centella asiatica* and *Bacopa monnieri* in animals. *Journal of Natural Remedies*.

[15] Wijeweera P, Arnason JT, Koszycki D, Merali Z (2006). Evaluation of anxiolytic properties of Gotukola—(*Centella asiatica*) extracts and asiaticoside in rat behavioral models. *Phytomedicine*.

[16] Wang XS, Dong Q, Zuo JP, Fang JN (2003). Structure and potential immunological activity of a pectin from *Centella asiatica* (L.) Urban. *Carbohydrate Research*.

[17] Gnanapragasam A, Kumar Ebenezar K, Sathish V, Govindaraju P, Devaki T (2004). Protective effect of *Centella asiatica* on antioxidant tissue defense system against adriamycin induced cardiomyopathy in rats. *Life Sciences*.

[18] Raghavendra M, Maiti R, Kumar S, Trigunayat A, Mitra S, Acharya S (2009). Role of *Centella asiatica* on cerebral post-ischemic reperfusion and long-term hypoperfusion in rats. *International Journal of Green Pharmacy*.

[19] Venu Gopal Rao ML, Mastan SA (2007). Antidiabetic effects of methanolic extract of *Centella asiatica* (Linn.) on induced hyperglycemic rats. *Biosciences Biotechnology Research Asia*.

[20] Lee YS, Jin DQ, Kwon EJ (2002). Asiatic acid, a triterpene, induces apoptosis through intracellular Ca^2+^ release and enhanced expression of p53 in HepG2 human hepatoma cells. *Cancer Letters*.

[21] Bunpo P, Kataoka K, Arimochi H (2004). Inhibitory effects of *Centella asiatica* on azoxymethane-induced aberrant crypt focus formation and carcinogenesis in the intestines of F344 rats. *Food and Chemical Toxicology*.

[22] Yoosook C, Bunyapraphatsara N, Boonyakiat Y, Kantasuk C (2000). Anti-herpes simplex virus activities of crude water extracts of Thai medicinal plants. *Phytomedicine*.

[23] Zaidan MR, Noor Rain A, Badrul AR, Adlin A, Norazah A, Zakiah I (2005). *In vitro* screening of five local medicinal plants for antibacterial activity using disc diffusion method. *Tropical Biomedicine*.

[24] Senthilkumar N, Varma P, Gurusubramanian G (2009). Larvicidal and adulticidal activities of some medicinal plants against the Malarial Vector, *Anopheles stephensi* (Liston). *Parasitology Research*.

[25] Naz E, Ahmad M (2009). Evaluation of five indigenous medicinal plants of Sindh, Pakistan for their antifungal potential. *Pakistan Journal of Scientific and Industrial Research*.

[26] Hamid AA, Shah Z, Muse R, Mohamed S (2002). Characterisation of antioxidative activities of various extracts of *Centella asiatica* (L) Urban. *Food Chemistry*.

[27] Jayashree G, Kurup Muraleedhara G, Sudarslal S, Jacob VB (2003). Anti-oxidant activity of *Centella asiatica* on lymphoma-bearing mice. *Fitoterapia*.

[28] Bajpai M, Pande A, Tewari SK, Prakash D (2005). Phenolic contents and antioxidant activity of some food and medicinal plants. *International Journal of Food Sciences and Nutrition*.

[29] Chaudhuri S, Ghosh S, Chakraborty T (1978). Use of a common Indian herb “Mandukaparni” in the treatment of leprosy. (Preliminary report). *Journal of the Indian Medical Association*.

[30] Pointel JP, Boccalon H, Cloarec M (1987). Titrated extract of *Centella asiatica* (TECA) in the treatment of venous insufficiency of the lower limbs. *Angiology*.

[31] Cesarone MR, Belcaro G, Rulo A (2001). Microcirculatory effects of total triterpenic fraction of *Centella asiatica* in chronic venous hypertension: measurement by laser Doppler, TcPo2-co2, and leg volumetry. *Angiology*.

[32] Williamson E, Williamson E (2002). *Centella asiatica* (L.) Urb. *Major Herbs of Ayurveda*.

[33] Pan J, Kai G, Yuan C, Zhou B, Jin R, Yuan Y (2007). Separation and determination of madecassic acid in extracts of *Centella asiatica* using high performance liquid chromatography with *β*-cyclodextrin as mobile phase additive. *Chinese Journal of Chromatography*.

[34] Sahu NP, Roy SK, Mahato SB (1989). Spectroscopic determination of structures of triterpenoid trisaccharides from *Centella asiatica*. *Phytochemistry*.

[35] Kuroda M, Mimaki Y, Harada H, Sakagami H, Sashida Y (2001). Five new triterpene glycosides from *Centella asiatica*. *Natural Medicines*.

[36] Siddiqui BS, Aslam H, Ali ST, Khan S, Begum S (2007). Chemical constituents of *Centella asiatica*. *Journal of Asian Natural Products Research*.

[37] Shukla YN, Srivastava R, Tripathi AK, Prajapati V (2000). Characterization of an ursane triterpenoid from *Centella asiatica* with growth inhibitory activity against *Spilarctia obliqua*. *Pharmaceutical Biology*.

[38] Matsuda H, Morikawa T, Ueda H, Yoshikawa M (2001). Medicinal foodstuffs. XXVI. Inhibitors of aldose reductase and new triterpene and its oligoglycoside, centellasapogenol A and centellasaponin A, from *Centella asiatica* (Gotu Kola). *Heterocycles*.

[39] Matsuda H, Morikawa T, Ueda H, Yoshikawa M (2001). Medicinal foodstuffs. XXVII. Saponin constituents of gotu kola (2): structures of new ursane- and oleanane-type triterpene oligoglycosides, centellasaponins B, C, and D, from *Centella asiatica* cultivated in Sri Lanka. *Chemical and Pharmaceutical Bulletin*.

[40] Yoshida M, Fuchigami M, Nagao T (2005). Antiproliferative constituents from umbelliferae plants VII. Active triterpenes and rosmarinic acid from *Centella asiatica*. *Biological and Pharmaceutical Bulletin*.

[41] Rumalla CS, Ali Z, Weerasooriya AD, Smillie TJ, Khan IA (2010). Two new triterpene glycosides from *Centella asiatica*. *Planta Medica*.

[42] Subban R, Veerakumar A, Manimaran R, Hashim KM, Balachandran I (2008). Two new flavonoids from *Centella asiatica* (Linn.). *Journal of Natural Medicines*.

[43] Wang XS, Duan JY, Fang JN (2004). Structural features of a polysaccharide from *Centella asiatica*. *Chinese Chemical Letters*.

[44] Govindan G, Sambandan TG, Govindan M (2007). A bioactive polyacetylene compound isolated from *Centella asiatica*. *Planta Medica*.

[45] Srivastava R, Shukla YN (1996). Some chemical constituents from *Centella asiatica*. *Indian Drugs*.

[46] Sondhi N, Bhardwaj R, Kaur S, Chandel M, Kumar N, Singh B (2010). Inhibition of H_2_O_2_-induced DNA damage in single cell gel electrophoresis assay (comet assay) by castasterone isolated from leaves of *Centella asiatica*. *Health*.

[47] Suntornsuk L, Anurukvorakun O (2005). Precision improvement for the analysis of flavonoids in selected Thai plants by capillary zone electrophoresis. *Electrophoresis*.

[48] Erdogan Orhan I, Atasu E, Senol FS High-throughput bioactivity screening of the Southeast Asian vegetable *Centella asiatica* (L.) Urban (gotu kola) and its phytochemical analysis.

[49] Asakawa Y, Matsuda R, Takemoto T (1982). Mono- and sesquiterpenoids from *Hydrocotyle* and *Centella* species. *Phytochemistry*.

[50] Oyedeji OA, Afolayan AJ (2005). Chemical composition and antibacterial activity of the essential oil of Centella asiatica growing in South Africa. *Pharmaceutical Biology*.

[51] Wongfhun P, Gordon MH, Apichartsrangkoon A (2010). Flavour characterisation of fresh and processed pennywort (*Centella asiatica* L.) juices. *Food Chemistry*.

[52] Howes MJR, Houghton PJ (2003). Plants used in Chinese and Indian traditional medicine for improvement of memory and cognitive function. *Pharmacology Biochemistry and Behavior*.

[53] Orhan G, Orhan I, Şener B (2006). Recent developments in natural and synthetic drug research for Alzheimer’s Disease. *Letters in Drug Design and Discovery*.

[54] Mukherjee PK, Kumar V, Houghton PJ (2007). Screening of Indian medicinal plants for acetylcholinesterase inhibitory activity. *Phytotherapy Research*.

[55] Khan MTH (2007). Molecular design of tyrosinase inhibitors: a critical review of promising novel inhibitors from synthetic origins. *Pure and Applied Chemistry*.

[56] Awad R, Levac D, Cybulska P, Merali Z, Trudeau VL, Arnason JT (2007). Effects of traditionally used anxiolytic botanicals on enzymes of the *γ*-aminobutyric acid (GABA) system. *Canadian Journal of Physiology and Pharmacology*.

[57] Xu Y, Cao Z, Khan I, Luo Y (2008). Gotu Kola (*Centella Asiatica*) extract enhances phosphorylation of cyclic AMP response element binding protein in neuroblastoma cells expressing amyloid beta peptide. *Journal of Alzheimer’s Disease*.

[58] Ramesh BN, Indi SS, Rao KSJ (2010). Studies to understand the effect of *Centella asiatica* on A*β*(42) aggregation *in vitro*. *Current Trends in Biotechnology and Pharmacy*.

[59] Barbosa NR, Pittella F, Gattaz WF (2008). Centella asiatica water extract inhibits iPLA2 and cPLA2 activities in rat cerebellum. *Phytomedicine*.

[60] Soumyanath A, Zhong YP, Gold SA (2005). *Centella asiatica* accelerates nerve regeneration upon oral administration and contains multiple active fractions increasing neurite elongation *in-vitro*. *Journal of Pharmacy and Pharmacology*.

[61] Lee MK, Kim SR, Sung SH (2000). Asiatic acid derivatives protect cultured cortical neurons from glutamate-induced excitotoxicity. *Research Communications in Molecular Pathology and Pharmacology*.

[62] Veerendra Kumar MH, Gupta YK (2002). Effect of different extracts of *Centella asiatica* on cognition and markers of oxidative stress in rats. *Journal of Ethnopharmacology*.

[63] Veerendra Kumar MH, Gupta YK (2003). Effect of *Centella asiatica* on cognition and oxidative stress in an intracerebroventricular streptozotocin model of Alzheimer’s disease in rats. *Clinical and Experimental Pharmacology and Physiology*.

[64] Gupta YK, Kumar MHV, Srivastava AK (2003). Effect of *Centella asiatica* on pentylenetetrazole-induced kindling, cognition and oxidative stress in rats. *Pharmacology Biochemistry and Behavior*.

[65] Rao SB, Chetana M, Uma Devi P (2005). Centella asiatica treatment during postnatal period enhances learning and memory in mice. *Physiology and Behavior*.

[66] Gadahad MR, Rao M, Rao G (2008). Enhancement of hippocampal CA3 neuronal dendritic arborization by *Centella asiatica* (Linn) fresh leaf extract treatment in adult rats. *Journal of the Chinese Medical Association*.

[67] Rao KGM, Muddanna Rao S, Gurumadhva Rao S (2009). Enhancement of amygdaloid neuronal dendritic arborization by fresh leaf juice of *Centella asiatica* (Linn) during growth spurt period in rats. *Evidence-based Complementary and Alternative Medicine*.

[68] Dhanasekaran M, Holcomb LA, Hitt AR (2009). Centella asiatica extract selectively decreases amyloid *β* levels in hippocampus of alzheimer’s disease animal model. *Phytotherapy Research*.

[69] Shinomol GK, Muralidhara K (2008). Effect of *Centella asiatica* leaf powder on oxidative markers in brain regions of prepubertal mice *in vivo* and its *in vitro* efficacy to ameliorate 3-NPA-induced oxidative stress in mitochondria. *Phytomedicine*.

[70] Haleagrahara N, Ponnusamy K (2010). Neuroprotective effect of *Centella asiatica* extract (CAE) on experimentally induced parkinsonism in aged Sprague-Dawley rats. *Journal of Toxicological Sciences*.

[71] Sudha S, Kumaresan S, Amit A, David J, Venkataraman BV (2002). Anti-convulsant activity of different extracts of *Centella asiatica* and Bacopa monnieri in animals. *Journal of Natural Remedies*.

[72] Ganachari MS, Veeresh Babu SV, Katare SS (2004). Neuropharmacology of an extract derived from *Centella asiatica*. *Pharmaceutical Biology*.

[73] Vattanajun A, Watanabe H, Tantisira MH, Tantisira B (2005). Isobolographically additive anticonvulsant activity between *Centella asiatica's* ethyl acetate fraction and some antiepileptic drugs. *Journal of the Medical Association of Thailand*.

[74] De Lucia R, Sertie JAA, Camargo EA, Panizza S (1997). Pharmacological and toxicological studies on *Centella asiatica* extract. *Fitoterapia*.

[75] Visweswari G, Siva Prasad K, Lokanatha V, Rajendra W (2010). The antiepileptic effect of Centella asiatica on the activities of Na^+^ /K^+^ , Mg^2+^ and Ca^2+^ -ATPases in rat brain during pentylenetetrazol-induced epilepsy. *Indian Journal of Pharmacology*.

[76] Wijeweera P, Arnason JT, Koszycki D, Merali Z (2006). Evaluation of anxiolytic properties of Gotukola—(*Centella asiatica*) extracts and asiaticoside in rat behavioral models. *Phytomedicine*.

[77] Ramaswamy AS, Pariyaswami SM, Basu N (1970). Pharmacological studies on *Centella asiatica*.Linn. *Indian Journal of Medicinal Research*.

[78] Bajpai M, Pande A, Tewari SK, Prakash D (2005). Phenolic contents and antioxidant activity of some food and medicinal plants. *International Journal of Food Sciences and Nutrition*.

[79] Hussin M, Abdul-Hamid A, Mohamad S, Saari N, Ismail M, Bejo MH (2007). Protective effect of *Centella asiatica* extract and powder on oxidative stress in rats. *Food Chemistry*.

[80] Flora SJS, Gupta R (2007). Beneficial effects of *Centella asiatica* aqueous extract against arsenic-induced oxidative stress and essential metal status in rats. *Phytotherapy Research*.

[81] Krishnamurthy RG, Senut MC, Zemke D (2009). Asiatic acid, a pentacyclic triterpene from *Centella asiatica*, is neuroprotective in a mouse model of focal cerebral ischemia. *Journal of Neuroscience Research*.

[82] Kumar A, Dogra S, Prakash A (2009). Neuroprotective effects of *Centella asiatica* against Intracerebroventricular colchicine-induced cognitive impairment and oxidative stress. *International Journal of Alzheimer’s Disease*.

[83] Xu CL, Wang QZ, Sun LM (2012). Asiaticoside: attenuation of neurotoxicity induced by MPTP in a rat model of Parkinsonism *via* maintaining redox balance and up-regulating the ratio of Bcl-2/Bax. *Pharmacology Biochemistry and Behavior*.

[84] Chen Y, Han T, Rui Y, Yin M, Qin L, Zheng H (2005). Effects of total triterpenes of *Centella asiatica* on the corticosterone levels in serum and contents of monoamine in depression rat brain. *Zhong yao Cai*.

[85] Appa Rao MVR, Srinivasan K, Koteswara Rao T (1977). The effect of *Centella asiatica* on the general mental ability of mentally retarded children. *Indian Journal of Psychiatry*.

[86] Wattanathorn J, Mator L, Muchimapura S (2008). Positive modulation of cognition and mood in the healthy elderly volunteer following the administration of *Centella asiatica*. *Journal of Ethnopharmacology*.

[87] Dev RDO, Mohamed S, Hambali Z, Samah BA (2009). Comparison on cognitive effects of *Centella asiatica* in healthy middle age female and male volunteers. *European Journal of Scientific Research*.

[88] Tiwari S, Singh S, Patwardhan K, Gehlot S, Gambhir IS (2008). Effect of *Centella asiatica* on mild cognitive impairment (MCI) and other common age-related clinical problems. *Digest Journal of Nanomaterials and Biostructures*.

[89] Bilbao I, Aguirre A, Zabala R, Gonzalez R, Raton J, Perez JLD (1995). Allergic contact dermatitis from butoxyethyl nicotinic acid and *Centella asiatica* extract. *Contact Dermatitis*.

[90] Gonzalo-Garijo MA, Revenga-Arranz F, Bobadilla-Gonzalez P (1996). Allergic contact dermatitis due to *Centella asiatica*: a new case. *Allergy and Immunopathology*.

[91] Gomes J, Pereira T, Vilarinho C, Duarte MDL, Brito C (2010). Contact dermatitis due to *Centella asiatica*. *Contact Dermatitis*.

[92] Singh B, Rastogi RP (1968). Chemical examination of *Centella asiatica* linn-III. Constitution of brahmic acid. *Phytochemistry*.

[93] Newall CA, Anderson LA, Phillipson JD (1996). *Herbal Medicines*.

[94] Dutta T, Basu UP (1968). Crude extract of *Centella asiatica* and products derived from its glycosides as oral antifertility agents.. *Indian Journal of Experimental Biology*.

